# Multidisciplinary Tumor Board Documentation: Assessing its Frequency, Quality, and Impact

**DOI:** 10.1245/s10434-026-19901-w

**Published:** 2026-06-03

**Authors:** Andrea N. Riner, Wilson Alobuia, Amanda Walsh, Valerie P. Grignol, Carlo M. Contreras, Timothy M. Pawlik, Susan Tsai, Jordan M. Cloyd

**Affiliations:** 1 Department of Surgery, Division of Surgical Oncology, The Ohio State University Wexner Medical Center, Columbus, OH USA; 2 The James Comprehensive Cancer Center, Columbus, OH USA

**Keywords:** Multidisciplinary tumor board, Documentation, Standards, Guidelines

## Abstract

**Background:**

Multidisciplinary tumor board (TB) meetings are used to generate consensus recommendations for the diagnosis and management of patients with cancer, yet no documentation standards exist. Since lack of documentation could contribute to patient outcomes, we aimed to characterize TB documentation practices at a single institution.

**Patients and Methods:**

A retrospective audit of patients discussed at six TBs at a National Cancer Institute (NCI)-designated comprehensive cancer center from January 2024 to June 2024 was conducted. The presence and quality of TB documentation in the medical records were recorded. Associations with clinicodemographic variables and patient outcomes were assessed.

**Results:**

Among 991 patients (307 liver, 235 breast, 166 colorectal, 153 pancreas, 110 cutaneous, 20 gastroesophageal), the median age was 63 years, 84.5% were white, 52.5% were female, and 65.7% had locoregional disease. Documentation was present in only 51.7% but varied by disease site (5.5% for breast to 97.0% for colorectal, *p* < 0.001). Among patients with documentation, 44.7% of notes were unstructured with variable content: 99.2% documented consensus recommendations, 58.0% listed the information reviewed, 29.3% listed attendees’ specialties, 11.3% documented the reason for discussion, and 15.8% documented clinical trial eligibility. In total, 55.7% of notes documented if patients were notified of the recommendations, most commonly via phone (71.8%).

**Conclusions:**

In this retrospective study, the presence and quality of documentation in the medical records are highly variable following discussion at a multidisciplinary TB meeting and could serve as a surrogate for high-quality multidisciplinary cancer care. Developing standards for TB documentation may improve accurate and timely dissemination of consensus recommendations and delivery of complex cancer care.

**Supplementary Information:**

The online version contains supplementary material available at 10.1245/s10434-026-19901-w.

Multidisciplinary cancer care is the cornerstone of contemporary oncology. Tumor board (TB) meetings are routinely held at most institutions globally as a common method of facilitating multidisciplinary cancer care.^1^ Participants often include medical and radiation oncologists, surgeons, radiologists, pathologists, and ancillary staff members such as research nurses and administrative coordinators. These meetings serve as a forum to review diagnoses, patient factors, tumor biology, disease stage, treatment options, clinical trial eligibility, and to reach a consensus for management decisions, in concordance with current guidelines and standards of care. Prior research has found that TB meetings frequently lead to a change in clinical management and can be associated with improved guideline-concordant care, increased utilization of targeted therapies, increased clinical trial enrollment, and better overall patient outcomes.^2^^–^^11^

There are over 1400 cancer programs accredited by The American College of Surgeons’ Commission on Cancer (ACS CoC), which requires compliance with specific standards to obtain this designation.^12^ The ACS CoC’s accreditation standards, Optimal Resources for Cancer Care (2020 Standards), define a minimum of 15% of cases be presented at TB and outline key elements for discussion at TB meetings.^13^ Yet, no specific standards (from ACS CoC, National Comprehensive Cancer Network (NCCN), or American Society of Clinical Oncology (ASCO) exist for how to document the results of a TB meeting or even whether formal documentation is required. Previous efforts at developing standards and/or checklists for multidisciplinary TBs have also failed to include recommendations on documentation.^14^^,^^15^ Such documentation might include which attendees were present, the clinical information on which management decisions were based, final consensus recommendations, and whether the patient was notified of the recommendations. More recently, specialized accreditation organizations, such as the National Accreditation Program for Breast Centers (NAPBC)^16^ and National Accreditation for Rectal Cancer (NAPRC),^17^ have developed disease-specific recommendations, including documentation standards, yet adherence to these recommendations is unclear.

Given the importance of multidisciplinary TBs in contemporary oncology and the lack of best practice standards for TB documentation, the purpose of this study was to characterize documentation practices at a single institution across diverse cancer specialties, including its prevalence, quality, and association with patient outcomes. These data are necessary to inform future work aimed at standardizing best practices for TB documentation, with the goal of facilitating the timely and accurate dissemination of multidisciplinary specialty oncology recommendations.

## Patients and Methods

This study is a noninterventional, retrospective review of six specialty multidisciplinary TB meetings (breast, colorectal, cutaneous, liver, pancreas, and gastroesophageal) performed at a single large comprehensive cancer center in the USA. These disease sites were chosen to represent the diversity of TB practices at our institution; all are conducted weekly and via virtual meetings. Some TBs have a designated administrative navigator (colorectal, liver, breast, gastroesophageal), while others have a physician champion alone (pancreas, cutaneous). All patients submitted for TB review during a 6-month period (1 January 2024 to 30 June 2024) were included. Exclusion criteria included patients who were on TB lists without any evidence that they had been formally discussed. We assessed cancer treatment decision-making and oncologic follow-up to capture their adherence to the outlined treatment plan and clinical status.

Through chart review, the following variables were collected: age, sex, race, ethnicity, cancer type/histology, date of diagnosis (preferentially determined by date of biopsy, followed by imaging date), date of TB discussion, clinical stage (metastatic, locoregional, benign/premalignant), new diagnosis versus established patient, specialty of referring provider, TB question to be discussed, discussion of clinical trial eligibility and subsequent enrollment in a study, date of disease progression, and date of last follow up with vital status. The study period was chosen to ensure that all patients had at least 12 months’ follow-up data.

Presence of TB documentation was defined as a separate note within the medical record detailing the discussion but not restricted by author: notes could be written by the primary provider, nurse navigator, or other individual. Reference to TB discussion embedded within other clinical notes was not classified as adequate TB documentation for the purposes of this study. When a TB note was present, TB documentation quality was assessed by recording documentation of the following variables: structure of the note (structured, semi-structured, unstructured), specialty of attendees present, clinical information reviewed, final consensus recommendations, clinical research study eligibility, patient notification (within 2 business days) along with method of notification, and whether TB recommendations were followed, with rationale if not followed.

### Statistical Analysis

Descriptive statistics were used to compare baseline characteristics between patients with documentation and those without documentation. Continuous variables were compared using the Wilcoxon rank-sum test, and categorical variables using *χ*^2^ or Fisher’s exact tests. Survival was estimated using the Kaplan–Meier method and log-rank test for differences in statistical significance. Multivariable Cox proportional hazards models were constructed to evaluate the independent association of documentation status on overall survival (OS) and progression-free survival (PFS), adjusting for relevant variables selected for their clinical significance or statistical significance on multivariate regression. Proportional hazards assumptions were verified using Schoenfeld residuals and log-log survival plots. Variables violating the assumption were modeled with time-varying effects using an extended Cox model, where the covariate was interacted with a function of time. This extended Cox model allows the hazard ratio associated with the covariate to change as a function of time. All analyses were performed using Stata 15.1 (College Station, TX, USA) and statistical significance was defined as *p* < 0.05 for a two‐sided test. This study was approved by The Ohio State University Cancer Institutional Review Board (no. 2024C0196).

## Results

### Patient Characteristics

Among 991 patients (307 liver, 235 breast, 166 colorectal, 153 pancreas, 110 cutaneous, 20 gastroesophageal), the median age was 63 years (interquartile range [IQR] 51–75 years) (Table 1). Most patients identified as white (84.5%), nonHispanic (96.0%), and were female (52.2%). Most had locoregional cancer staging (65.7%), whereas 17.3% of patients had metastatic disease and 17.1% of patients had benign or premalignant conditions. Most patients discussed at TB were being discussed as a new diagnosis (64.9%). During the 12 months of follow-up, 30.5% of patients had disease progression (median time to recurrence was 158.5 days (IQR 75.5–271.5 days), with 17.7% dying during the follow-up period (median time to death was 474 days, IQR 397–526 days).

**Table 1:** Patient and TB characteristics

	Total, no. (%)	TB documentation present, no. (%)	No TB documentation present, no. (%)	*p*-Value
Age (years)				**0.037**
Median (IQR)		64 (55–72)	63 (49–71)	
Sex				**< 0.001**
Male	474 (47.8)	321 (62.7)	153 (31.9)	
Female	517 (52.2)	191 (37.3)	326 (68.1)	
Race				0.415
White/Caucasian	837 (84.5)	425 (83.0)	412 (86.0)	
Black/African American	106 (10.7)	61 (11.9)	45 (9.4)	
Asian or Pacific Islander	16 (1.6)	7 (1.4)	9 (1.9)	
American Indian/Alaskan Native	2 (0.2)	2 (0.4)	0 (0)	
More than one race	4 (0.4)	3 (0.6)	1 (0.2)	
Other	21 (2.1)	10 (2.0)	11 (2.3)	
Ethnicity				0.510
Non-Hispanic	951 (96.0)	490 (95.7)	461 (96.2)	
Hispanic	24 (2.4)	14 (2.7)	10 (2.1)	
TB Disease Site				**< 0.001**
Breast	235 (23.7)	13 (2.5)	222 (46.3)	
Colorectal	166 (16.8)	161 (31.4)	5 (1.0)	
Cutaneous	110 (11.1)	10 (2.0)	100 (20.9)	
Gastroesophageal	20 (2.0)	3 (0.6)	17 (3.5)	
Liver	307 (31.0)	270 (52.7)	37 (7.7)	
Pancreas	153 (15.4)	55 (10.7)	98 (20.5)	
Stage				**< 0.001**
Benign/premalignant	169 (17.1)	111 (21.7)	58 (12.1)	
Locoregional	651 (65.7)	332 (64.8)	319 (66.6)	
Metastatic	171 (17.3)	69 (13.5)	102 (21.3)	
New patient				**< 0.001**
Yes	643 (64.9)	303 (59.2)	340 (71.0)	
No	348 (35.1)	209 (40.8)	139 (29.0)	
Recurrence/progression				**0.004**
Yes	302 (30.5)	177 (34.6)	125 (26.1)	
No	688 (69.5)	335 (65.4)	353 (73.7)	
Median time to recurrence/progression in days (IQR)		157 (90–273)	153.5 (57–254)	0.468
Alive at 12 months				0.945
Yes	816 (82.4)	90 (17.6)	85 (17.7)	
No	175 (17.7)	422 (82.4)	394 (82.3)	
Median time to death in days (IQR)		463 (379–530)	476 (398–521)	0.892

Bold = *p*-value < 0.05

### Presence and Quality of Documentation

TB documentation within the medical record was present approximately half the time (512 patients, 51.7%) but varied significantly by disease site (e.g., 5.5% breast to 97.0% colorectal, *p* < 0.001) (Table 1). Other factors associated with TB documentation included older age (64 vs. 63 years, *p* = 0.037), sex (62.7% male versus 37.3% female, *p* < 0.001), timing of presentation (new diagnosis versus follow-up, *p* < 0.001), disease stage (*p* < 0.001), and disease progression (*p* = 0.004). Presence of TB documentation was not associated with enrollment in a clinical trial (4.7% of those with documentation versus 3.3% of those without documentation, *p* = 0.282). Among patients with documentation noting consensus recommendations (*n* = 508), TB recommendations were followed 88.4% of the time. The most common reasons for nonadherence to consensus recommendations included patient preference (38.6%), disease progression (29.8%), lost to follow-up (8.8%), change in treatment plan (7.0%), patient condition (5.3%), technical issues (5.3%), and insurance denial (1.8%).

Measures of documentation quality are presented in Table 2. Among patients with documentation present in the medical record, 44.7% of notes were unstructured and the contents within the notes were highly variable: 99.2% documented final consensus recommendations, 58.0% listed the information reviewed, 29.3% listed the specialty of attendees, and 15.8% documented clinical trial eligibility. Overall, 55.7% of notes documented if patients were notified of the recommendations, most commonly via phone (71.8%), less often electronically (9.9%) or in-person (10.6%).

**Table 2:** Details on quality of TB documentation among those with a note documented in the medical record

	Total, no. (%)
Documentation present	
Yes	512 (51.7)
No	479 (48.3)
Documentation type	
Structured	167 (32.6)
Semi-structured	116 (22.7)
Unstructured	229 (44.7)
Attendee specialty	
Yes	150 (29.3)
No	362 (70.7)
TB question	
Yes	58 (11.3)
No	454 (88.7)
TB question type	
New patient	179 (35.4)
Review treatment plan	145 (28.7)
Imaging review	97(19.2)
Assess treatment response	50 (9.9)
Follow-up question	19 (3.8)
Path review	16 (3.2)
Information reviewed	
Yes	297 (58.0)
No	215 (42.0)
Trial eligibility	
Yes	80 (15.8)
No	427 (84.2)
Consensus recommendations	
Yes	508 (99.2)
No	4 (0.8)
Patient notified	
Yes	284 (55.7)
No	226 (44.3)
Method of notification	
In person	30 (10.6)
EMR message	28 (9.9)
Phone	204 (71.8)
Telehealth	4 (1.4)
Unknown	18 (6.3)

### Exploratory Survival Analysis

**Fig. 1:**
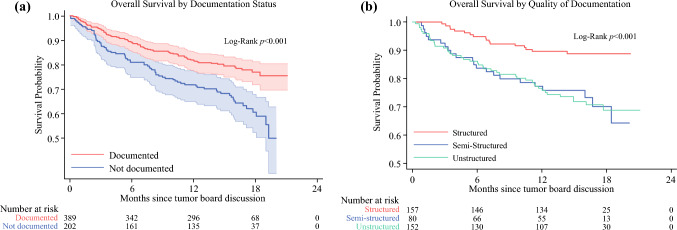
**A** Overall survival by presence of TB documentation (HR 0.61, 95% CI 0.40–0.95; *p* = 0.029); **B** overall survival by quality of TB documentation (structured documentation: HR 0.42, 95% CI 0.23–0.75, *p* = 0.003)

**Fig. 2:**
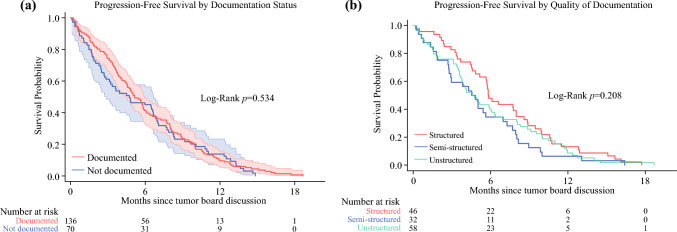
**A** Progression-free survival by presence of TB documentation (HR 0.86, 95% CI 0.54–1.36; *p* = 0.526); **B** progression-free survival by quality of TB documentation (structured documentation: HR 0.79, 95% CI 0.53–1.20; *p* = 0.281)

After excluding patients with breast cancer owing to nearly universal lack of documentation, both TB documentation (Fig. 1a; hazard ratio [HR] 0.61, 95% confidence interval [CI] 0.40–0.95; *p* = 0.029) and the use of structured notes were associated with improved overall survival (Fig. 1b; HR 0.42, 95% CI 0.23–0.75; *p* = 0.003). On the other hand, neither was associated with progression-free survival (Fig. 2a and b). On Cox regression analysis, neither overall TB documentation (HR 0.86, 95% CI 0.54–1.36; *p* = 0.526) nor structured documentation (HR 0.79, 95% CI 0.53–1.20; *p* = 0.281) were associated with PFS.

## Discussion

Multidisciplinary tumor boards are an integral component of contemporary oncology. While the literature is mixed on their overall efficacy, with some studies showing improved adherence to cancer guidelines, patient outcomes, and provider satisfaction, and others showing less clinical benefits, TBs are now routinely integrated into clinical cancer care at most centers around the world.^1^^–^^3^^,^^5^^,^^11^^,^^14^^,^^18^^,^^19^ Documentation following TB discussion may serve several purposes: enhanced communication for oncologic and non-oncologic providers, objective record for patients to access through the electronic health record, medical billing, medicolegal purposes, and recall of recommendations in the future. Despite the importance of TB documentation, little research has focused on this issue. Given the lack of best practice standards or even requirements for documentation, the purpose of this study was to characterize practices and the quality of TB documentation across a diverse sample of tumor boards at our institution. Notably, the presence and quality of TB documentation in the medical record were highly variable in our study, suggesting an opportunity to develop standards to improve the timely and accurate dissemination of multidisciplinary TB recommendations.

The reasons for relatively low rates of TB documentation in our study remain unclear. Several factors, including patient age and sex, disease site, disease stage, and whether a patient was new to TB, were associated with lack of a TB note, yet the strongest factor was disease type. This suggests that distinct practices exist among tumor boards as well as the specialists who attend them. Owing to requirements for rectal and breast cancer accreditation, those two TB disease sites have more standardized, yet differing, practices at our institution. Specifically, the colorectal TB group utilized a standardized note template based on NAPRC guidance (Supplemental File), whereas the breast TB group documented their TB discussions outside of the medical record, which limits the accessibility of TB consensus recommendations to healthcare providers without access to this secure database. Note that the absence of TB documentation within the medical record does not imply a lack of robust or meaningful discussion; yet it prevents the dissemination of these recommendations to persons who were not present at the meeting (including oncology providers, other health care team members, patients, and even the primary provider in the future who may or may not recall the specifics of the discussion). Overall, documentation practices at our institution were highly variable and essentially voluntarily dependent on the practice of individual providers. Future research is needed to understand the barriers and facilitators to comprehensive, high-quality TB documentation within the medical record.

The results of our study also highlight the need to develop standards on the content of TB notes. Indeed, even among those patients where notes were included in the medical record, the structure and quality of these notes varied significantly. For example, many notes were unstructured and lacked specific pertinent content, including the specialties of attendees, the questions to be answered, the information reviewed, and whether patients were notified of the consensus recommendations. Future research could develop standards for the optimal elements of a TB documentation note, especially since not all elements included in our study are necessarily of equal importance. Structured templates may be a helpful tool to improve the fidelity of clinically meaningful information documented in the medical record and have the potential to guide or standardize patient presentations during TB meetings.^15,20^ In fact, previous research has shown that synoptic reports have significantly enhanced the quality and completeness of reporting clinically relevant information for pathologic, radiologic, and surgical documentation.^21^^–^^27^ Naturally, there is increasing interest in the use of artificial intelligence to facilitate TB meetings and improve dissemination and communication, although best-practice standards are still necessary to train these models.^28^^,^^29^

While the importance of TB documentation to multidisciplinary cancer care may be clear, future research should evaluate potential barriers and disadvantages while developing and implementing standards. For example, requirements for TB documentation, if not paired with solutions to help efficiency, can increase the administrative burden of TB meetings, which may limit their effective adoption. Future work may also aim to understand the medicolegal implications of TB documentation. While documentation is often prioritized to memorialize multidisciplinary support for a plan and protect against medicolegal risk, providers could have concerns about documentation if their diagnostic or treatment recommendations differ from the TB recommendations.

While the current study is novel and interesting, several limitations should be acknowledged. First, the study results are based on six TB meetings conducted weekly at a single high-volume cancer center and, therefore, may not be generalizable to all TB meetings. We attempted to select TBs that were representative of a wide range of practice styles, but acknowledge this remains a limited sample (indeed, even our own single institution has dozens of unique specialty TBs). Second, the study is a retrospective audit rather than a prospective cohort study, which can contribute to information and misclassification bias. As a single tertiary care cancer center, some of our patients are referred to us for second opinions but choose to seek cancer treatment closer to home, which could have limited our ability to capture accurate follow-up information regarding adherence to treatment recommendations, disease progression, and overall survival. Third, we defined TB documentation as a separate note in the medical record intentionally. Since TB consensus recommendations were sometimes embedded within other clinical notes and could still contain important information, our study findings could underestimate the true rate of documentation, yet it still highlights the heterogeneity of documentation practices. Finally, any differences in OS or PFS in our study are significantly limited owing to differences in clinical and demographic characteristics, both among the disease-specific TBs as well as between patients who do and do not have documentation. While the association between TB documentation and OS is interesting, it is likely heavily influenced by confounding and therefore should be considered hypothesis-generating only at this time. Nevertheless, future research should aim to further investigate whether TB documentation serves as a surrogate for high-quality multidisciplinary cancer care and, if multidisciplinary standards were successfully implemented, could improve patient outcomes.

In conclusion, this retrospective review of multidisciplinary TB meetings over 6 months at a large cancer center found that the presence and quality of TB meeting documentation in the medical record are highly variable. These findings highlight the need to develop standards for documenting and communicating the results of specialized oncology TB meetings in the medical record. Future research should focus on patient and provider perceptions of TB documentation in the medical record, optimizing efficiency and workflow related to documentation, and developing standards for high-quality TB documentation to improve the accurate and timely dissemination of consensus recommendations and the delivery of complex personalized cancer care.

## Supplementary Information

Below is the link to the electronic supplementary material.

Supplementary file1 (DOCX 18 KB)
