# Supplementary material for: Multidisciplinary Tumor Board Documentation: Assessing its Frequency, Quality, and Impact
Source: Ann Surg Oncol. 2026 Jun 3;33(9):8275–81. doi: 10.1245/s10434-026-19901-w (PMC13452832; doi:10.1245/s10434-026-19901-w)
Supplement: Supplementary file 1 [file 10434_2026_19901_MOESM1_ESM.docx]

**Supplemental Figure.**

**Rectal Cancer Tumor Board**

**Treatment Evaluation and Recommendation Summary**

**Date of Conference:** @TODAYDATE@

**Referring physician** (Treating physician): ***

**Colonoscopy reviewed**: {YES/NO:21290}

**Rigid sigmoidoscopy:** {YES/NO:21290}

**Pathology:** {tbcolopath2:46537}

**Pathology slides have been reviewed or report has been obtained**: {YES/NO:21290}

**MRI**

- Date: ***
- Reviewed: {YES/NO:21290}
- MRI stage{TCRCstaging:46125}
- {NCRCstaging:46126}
- {MCRCstaging:46127}

**CT chest:**

- Date:***
- Metastases: {YES/NO:21290}

**CT abdomen/pelvis:**

- Date: ***
- Metastases: {YES/NO:21290}

**PET**:

- Date: {None:33079}

**Other Imagining review:** {YES/NO:21290}

**Most recent CEA level:**

@LASTLABOSUSHORT(CEA,CEAPRESHAMA,CEANEWMETHOD)@

Tumor location in the rectum: {TB Colorectal Location:58412}

Sphincter involvement: {YES/NO:21290}

Pretreatment circumferential resection margin: {TB Pretreatment circumferential resection margin:58413}

**Pre-Treatment clinical stage:**

- {TCRCstaging:46125}
- {NCRCstaging:46126}
- {MCRCstaging:46127}

**Type of neoadjuvant therapy recommended:** {CRCTBoptions:45975}

**Duration of neoadjuvant therapy recommended:** ***

**Anticipated surgical treatment:** {APR/LAR:45974}

**Anticipated timing of surgical treatment:** ***

**Anticipated en bloc resection:** {YES/NO:20206}

**Anticipated metastasectomy:** {YES/NO:20206}

**Clinical Trial Candidate:** {YES/NO:21290}

**Referrals to be placed**:{TB Referral Orders:58414}

**Other Discussion:** ***

**Note to be sent to: *****

***This is the summary of the general discussion provided at tumor board conference. The final recommendations will be made by the primary health care team and the patient after discussing the benefits, risks and alternatives to the various treatment options.***
